# Physical Education as a Lifestyle Learning Environment for Neurobehavioral Well-Being: A Narrative Review

**DOI:** 10.3390/bs16081331

**Published:** 2026-08-03

**Authors:** Bingzheng Zhou, Ning Wang, Xiaobing Luo, Jing Zhao

**Affiliations:** 1 School of Sport Training, Tianjin University of Sport, Tianjin 301617, China; zhoubingzheng@tjus.edu.cn; 2 Basic Courses Department, Tianjin Sino-German University of Applied Sciences, Tianjin 300350, China; wangning@tsguas.edu.cn; 3 School of Physical Education and Sport, Central China Normal University, Wuhan 430079, China

**Keywords:** physical education, lifestyle learning environment, neurobehavioral well-being, lifestyle self-regulation, recovery-related awareness, nutrition-related awareness

## Abstract

Physical education (PE) is commonly justified through its contributions to physical fitness, motor skill development, sport participation, and health-enhancing physical activity. These aims remain important, but they do not fully capture PE’s potential as a curriculum context for lifestyle learning and neurobehavioral well-being. This narrative review used structured literature mapping and conceptual framework development to examine how PE may connect embodied movement experiences with motivation, stress and emotion regulation, self-efficacy, resilience, recovery-related awareness, and lifestyle self-regulation in adolescents and young adults. Literature searches were conducted iteratively in PubMed, Scopus, Web of Science Core Collection, SPORTDiscus, and ERIC, with the final search update completed on 20 June 2026. The synthesis identified a curriculum-to-lifestyle gap: existing evidence more often examines whether PE, physical activity, sport, or physical literacy is associated with health or psychological outcomes than how curriculum, teaching climate, reflection, assessment, and peer interaction may support transferable self-regulatory learning. The review therefore proposes an evidence-informed, curriculum-oriented conceptual framework linking PE design and pedagogy with proximal motivational, emotional, cognitive-regulatory, behavioral, and embodied processes. The framework does not imply that PE automatically improves mental health, directly produces neurobiological change, or replaces specialist care. Evidence is stronger for proximal educational processes than for sustained transfer beyond PE. Future research should test clearly specified curriculum mechanisms, implementation conditions, assessment approaches, and longitudinal transfer.

## 1. Introduction

Physical education (PE) has long been justified through its contributions to physical fitness, motor skill development, sport participation, and the promotion of health-enhancing physical activity. These aims remain important. PE is one of the few formal educational settings in which many students can encounter structured movement learning, health-related knowledge, and repeated opportunities for embodied participation. International guidance on quality physical education similarly emphasizes that PE should contribute not only to movement competence, but also to inclusive learning, lifelong participation, health, and personal development (UNESCO, 2015). Recent empirical work in a Romanian university sample further illustrates the relevance of health-related fitness within tertiary education. Using the European Fitness Badge, Geantă et al. (2025) identified variation in endurance, strength, coordination, and overall fitness according to activity profile, sex, age, academic major, and body composition, while also emphasizing the need for context-specific interpretation and targeted provision in university settings.

However, PE does not operate within a single global context. Its aims, curriculum organization, pedagogical traditions, assessment practices, institutional status, and student experiences vary across countries, cultures, educational systems, and age groups. The present review, therefore, addresses an international readership without assuming that one model of PE applies universally. Instead, it proposes an evidence-informed conceptual framework whose relevance, feasibility, and implementation should be interpreted and tested within specific educational and sociocultural contexts.

The cross-contextual basis of the framework derives from an international synthesis of literature on PE pedagogy, physical literacy, health literacy, motivation, self-regulation, and lifestyle-related learning, rather than from an assumption that any single national perspective represents PE globally. Across diverse settings, adolescents and young adults may encounter different combinations of psychological stress, motivational instability, sleep disruption, sedentary routines, social comparison, and uneven opportunities for health-related self-regulation. These patterns should not be understood as universal or identical across countries. Rather, they illustrate why PE may be reconsidered, where contextually appropriate, as a curriculum space in which students can interpret embodied experience and connect movement with lifestyle regulation and well-being (UNESCO, 2015).

A substantial body of review evidence has already examined links between physical activity, sport, PE provision, and mental health outcomes in young people. Systematic reviews suggest that school-based PE, physical activity, and sport provision may be associated with well-being, self-esteem, self-efficacy, depression, anxiety, and life satisfaction; however, the strength of these findings remains uneven across study designs, populations, intervention characteristics, and outcome measures (Rocliffe et al., 2024). Broader reviews of physical activity in children and adolescents report generally favorable associations with mental health, but they also caution that study quality, heterogeneity, and causal interpretation remain persistent limitations (Fu et al., 2025; Biddle & Asare, 2011). In tertiary education settings, physical literacy and physical activity have also been associated with mental well-being, resilience, life satisfaction, and lower psychological distress (Leung et al., 2025). This evidence base is valuable, but much of it remains organized around an outcome question: whether physical activity, PE, sport, or physical literacy is associated with psychological indicators. Less attention has been given to the more educationally specific question of how PE curriculum, pedagogy, and assessment may help students develop transferable forms of lifestyle self-regulation (Fu et al., 2025; Leung et al., 2025; Rocliffe et al., 2024).

This distinction matters because lifestyle behaviors relevant to neurobehavioral well-being are not isolated exposures. Physical activity, sedentary time, sleep, rest, nutrition-related practices, and recovery interact within daily routines, particularly during adolescence and young adulthood, when students gradually assume greater responsibility for organizing their own behaviors. Sleep is included in this review because sleep duration, timing, and quality may influence attention, mood, perceived effort, readiness for movement, and recovery. Rest is used more broadly to refer to periods of reduced physical or cognitive load, pacing, and opportunities for physiological and psychological restoration. Sleep and rest are therefore treated as related but non-identical components of recovery. Recent review evidence on physical activity, diet, and sleep in adolescents reinforces the need to view these lifestyle domains as connected rather than separate educational concerns (Cruz et al., 2024). Longitudinal evidence also indicates that physical activity does not follow a single developmental pattern from adolescence to young adulthood. Aira et al. identified distinct trajectories that included maintained activity, sustained inactivity, declining activity, and increasing activity between ages 15 and 19, highlighting the need to consider individual and contextual variation across this transition (Aira et al., 2021). Movement behavior guidelines for children and youth also integrate physical activity, sedentary behavior, and sleep within a 24-h framework, which reinforces the idea that student health depends on daily behavioral organization rather than on single-session activity exposure alone (Tremblay et al., 2016). For PE, this creates a specific educational opportunity. PE lessons, curricula, and teaching strategies may provide embodied entry points through which students learn to connect movement, fatigue, recovery, sleep, energy, mood, motivation, and everyday decision-making (Cruz et al., 2024; Tremblay et al., 2016; UNESCO, 2015).

To develop this broader argument, PE needs to be connected with behavioral-science concepts rather than treated only as a form of exercise exposure. Motivation is a central starting point. Self-Determination Theory proposes that autonomy, competence, and relatedness are basic psychological needs that shape motivation, development, and well-being (Ryan & Deci, 2000). In physical activity contexts, autonomous motivation has been associated with more sustained engagement and behavioral persistence (Teixeira et al., 2012).

In PE, need-supportive teaching refers to instructional practices that support students’ basic psychological needs for autonomy, competence, and relatedness. Such practices may include providing meaningful choice and rationale, acknowledging student perspectives, offering optimally challenging and differentiated tasks, giving constructive competence-related feedback, and fostering respectful and inclusive social interaction (Abula et al., 2020; Lonsdale et al., 2013; Ryan & Deci, 2000; Vasconcellos et al., 2020).

Original PE studies and intervention trials suggest that autonomy-supportive or need-supportive teaching may influence students’ perceived need support, autonomous motivation, engagement, and selected activity-related outcomes, although findings vary across interventions and contexts (Abula et al., 2020; Lonsdale et al., 2013). The review evidence provides broader support for these associations (Vasconcellos et al., 2020).

These findings point to a curriculum issue that is sometimes overlooked: the same movement task may carry different neurobehavioral meanings depending on whether students experience choice, competence, social connection, mastery, pressure, exclusion, or repeated failure (Ryan & Deci, 2000; Teixeira et al., 2012; Vasconcellos et al., 2020).

Stress and emotional regulation provide a second important pathway. Emotional regulation refers to the processes through which individuals influence which emotions they have, when they have them, and how these emotions are experienced and expressed (Gross, 2002). Adolescents and young adults routinely encounter academic, social, and identity-related stressors. PE may intensify these pressures when it is competitive, exclusionary, or narrowly evaluative; alternatively, it may provide structured experiences of coping, mastery, cooperation, and body awareness. Similarly, self-efficacy theory emphasizes that beliefs about one’s capability influence effort, persistence, and behavioral change (Bandura, 1977). When PE is designed around progressive challenge, meaningful feedback, and inclusive success experiences, it may contribute to students’ confidence in regulating effort, movement participation, and related lifestyle behaviors. Resilience research further suggests that adaptive development often emerges from ordinary processes embedded in relationships, structured opportunities, competence-building, and supportive environments (Masten, 2001). PE, therefore, has potential relevance not only as a physical activity setting, but also as a recurring educational context in which students may practice adaptive regulation through movement (Bandura, 1977; Gross, 2002; Masten, 2001).

This perspective is also grounded in PE curriculum and pedagogy scholarship. Health-based PE models have argued that PE should move beyond narrow fitness testing or sport technique instruction toward forms of learning that help students understand and enact health in contextually meaningful ways (Haerens et al., 2011). Related conceptual work has challenged the assumption that health in PE is a fixed outcome to be delivered, arguing instead that students can learn health through situated, embodied, and reflective experiences (Quennerstedt, 2019). From this viewpoint, PE becomes educationally significant when curriculum design, teaching climate, assessment, peer interaction, and reflective tasks are aligned with students’ developing capacity to interpret bodily experience and make health-related decisions (Haerens et al., 2011; Quennerstedt, 2019). This argument is consistent with broader health literacy frameworks, which define health literacy not as the passive receipt of information, but as the ability to access, understand, appraise, and use health information in everyday contexts (Sørensen et al., 2012). Taken together, these perspectives support a reframing of PE as a curriculum-based context for movement, health learning, and behavioral self-regulation (Haerens et al., 2011; Quennerstedt, 2019; Sørensen et al., 2012; UNESCO, 2015).

In this review, neurobehavioral well-being refers to the interacting motivational, emotional, cognitive-regulatory, behavioral, and embodied processes that shape students’ functioning and capacity for everyday self-regulation. These processes include motivation, self-efficacy, stress appraisal, emotion regulation, coping, resilience, self-monitoring, and behavioral planning. The term does not imply direct neurological change, clinical neurobehavioral treatment effects, or demonstrated alterations in brain structure, neural activity, neurochemistry, or biological markers resulting from ordinary PE participation.

The central question of this review is as follows: through which curriculum and pedagogical processes may PE help adolescents and young adults connect embodied movement experiences with transferable lifestyle self-regulation?

The primary aim is to develop an evidence-informed, curriculum-oriented conceptual framework that specifies proposed relationships among PE curriculum design, teaching climate, embodied experience, motivation, self-efficacy, stress and emotion regulation, recovery-related awareness, and transfer beyond PE lessons. The framework focuses on educational and behavioral processes rather than on direct neurological effects or clinical mental-health outcomes.

The review does not propose a standardized or empirically validated curriculum model. Instead, it uses the conceptual framework to derive curriculum and teaching principles that may be adapted to different school and university contexts. A secondary aim is to identify priority research questions, implementation conditions, and measurement approaches for testing and refining the proposed framework.

## 2. Narrative Review Methodology

### 2.1. Review Scope and Conceptual Domains

This article was conducted as a narrative review with structured literature mapping and conceptual framework development. The purpose was not to estimate pooled effects, evaluate the efficacy of a single intervention, or provide an exhaustive systematic synthesis of all research on physical education (PE), physical activity, or student mental health. Rather, the review aimed to integrate theoretically and educationally relevant evidence in order to develop an evidence-informed, curriculum-oriented conceptual framework explaining how PE may support lifestyle self-regulation and neurobehavioral well-being in adolescents and young adults. A narrative review design was considered appropriate because the review question spans heterogeneous theoretical, pedagogical, behavioral, and health-related studies that cannot be reduced to a single exposure, intervention, or outcome (Baumeister & Leary, 1997; Grant & Booth, 2009; Green et al., 2006; Snyder, 2019).

The review focused on adolescents and young adults participating in secondary-school, upper-secondary, college, university, or comparable structured movement- and wellness-oriented educational settings. At the tertiary level, this included relevant compulsory or elective activity courses, general education movement modules, wellness courses, and related educational provision, while recognizing that such formats are not available or labeled as PE in all national systems. The framework was intended to be context-sensitive rather than tied to one national curriculum system. Evidence from different educational systems was therefore considered when it informed transferable curriculum principles, while differences in PE provision, compulsory status, teacher preparation, assessment, and institutional context were acknowledged.

Eight interrelated literature domains were mapped: (1) PE curriculum and pedagogy; (2) PE, physical activity, sport, and student mental health; (3) physical literacy, health literacy, and mental health literacy; (4) motivation and Self-Determination Theory in PE and physical activity contexts; (5) stress regulation, emotion regulation, resilience, self-efficacy, and self-regulation; (6) sleep, recovery, and nutrition-related awareness; (7) school- and university-based health-promoting frameworks; and (8) narrative and conceptual review methodology.

The domains were developed through a hybrid, iterative process. The initial domain structure was informed by the central review question and by established theoretical and educational studies considered necessary to explain PE curriculum, student learning, behavioral regulation, and lifestyle transfer. During literature mapping, the domains were refined when recurrent constructs, overlapping terminology, and cross-domain relationships emerged. Closely related constructs were combined where they served similar explanatory functions, while additional domains were retained when they contributed a distinct element not captured elsewhere.

The domains were not derived from a pre-existing fixed framework, nor were they generated exclusively through inductive coding. They were also not formally weighted according to publication count, study design, effect size, or presumed importance. Such weighting was not appropriate because the review integrated heterogeneous theoretical, empirical, methodological, policy, and curriculum studies. Instead, each domain was evaluated according to its function within the synthesis: defining the evidence landscape, identifying the conceptual gap, explaining proposed mechanisms, informing curriculum implications, or supporting implementation and future research.

### 2.2. Search Strategy

Literature searches were conducted iteratively in PubMed, Scopus, Web of Science Core Collection, SPORTDiscus, and ERIC. PubMed was used primarily to identify literature concerning health, behavior, sleep, recovery, nutrition, and mental well-being. SPORTDiscus was used to identify PE, physical activity, sport pedagogy, physical literacy, and movement-related research. ERIC was used primarily for curriculum, educational practice, teaching, and school-based studies. Scopus and Web of Science Core Collection were used for multidisciplinary coverage, citation tracking, and cross-checking across educational, psychological, behavioral, and health-related fields. Targeted publisher searches and backward and forward citation tracking were used to identify additional theory papers, curriculum studies, official standards, and high-relevance empirical research. The final search update and reference verification were completed on 20 June 2026.

Search terms were organized into four concept blocks:

(1) educational context: “physical education,” “physical education curriculum,” “physical education pedagogy,” “physical education teaching,” “school physical education,” “university physical education,” or “wellness education”;
(2) population and setting: “adolescent*,” “youth,” “young adult*,” “student*,” “secondary school,” “college,” or “university”;
(3) lifestyle and behavioral processes: “physical literacy,” “health literacy,” “mental health literacy,” “motivation,” “self-determination,” “self-efficacy,” “self-regulation,” “stress regulation,” “emotion regulation,” “resilience,” “coping,” “sleep,” “recovery,” “nutrition education,” or “lifestyle”;
(4) outcomes and educational transfer: “well-being,” “mental health,” “engagement,” “behavior change,” “transfer,” “health behavior,” or “lifestyle regulation.”

Terms were combined using Boolean operators and adapted to the indexing conventions and search fields of each database. A representative search structure was:

(“physical education” OR “physical education curriculum” OR “physical education pedagogy”)

AND

(adolescent* OR “young adult*” OR student* OR university)

AND

(motivation OR self-regulation OR stress OR emotion regulation OR resilience OR sleep OR recovery OR nutrition OR lifestyle)

AND

(well-being OR “mental health” OR engagement OR transfer OR “health behavior”).

Because the review integrated several conceptually adjacent studies, supplementary searches were also conducted for individual theoretical constructs and established frameworks, including Self-Determination Theory, self-efficacy, self-regulated learning, physical literacy, health literacy, meaningful PE, health-based PE, and health-promoting educational settings. These targeted searches were used to identify foundational theoretical sources and representative empirical applications rather than to create independent systematic searches for each construct.

### 2.3. Eligibility and Literature Selection

Eligibility criteria were used to define the scope and conceptual boundaries of the narrative review. Publications were considered eligible when they met the following criteria:

(1) they addressed adolescents, young adults, students, or educational populations relevant to secondary or tertiary education;
(2) they examined PE, movement-based education, physical literacy, health-related learning, or a behavioral process directly relevant to the proposed framework;
(3) they contributed to at least one predefined manuscript function—mapping the existing review landscape, identifying a conceptual gap, supporting a proposed curriculum or neurobehavioral pathway, or informing curriculum design, implementation, assessment, or future research;
(4) they were peer-reviewed systematic reviews, scoping reviews, meta-analyses, umbrella reviews, conceptual or theory papers, measurement studies, or high-relevance empirical studies;
(5) the full text was available in English.

Authoritative organizational reports, policy documents, curriculum standards, and movement-behavior guidelines were also eligible when they provided definitions or recommendations directly relevant to quality PE, physical literacy, health literacy, student well-being, or health-promoting educational settings.

Publications were excluded or deprioritized when they focused primarily on clinical exercise therapy, rehabilitation, elite sport performance, older adults, neurological disease, specialized sleep medicine, individualized nutrition treatment, or clinical mental health intervention without a clear connection to PE curriculum, pedagogy, or educational transfer. Studies centered exclusively on acute physiological or neurobiological responses were also outside the principal scope unless they informed the interpretation of embodied learning or behavioral regulation without being used to imply direct neurological effects of ordinary PE participation.

Selection proceeded through iterative title, abstract, and full-text assessment for conceptual relevance. At the title and abstract stage, publications were retained when their population, educational setting, construct, or outcome appeared relevant to at least one review domain. Full texts were then assessed to determine whether they added a distinct theoretical, empirical, methodological, or translational contribution. Publications were excluded at this stage when PE or education was only incidental, when the population or setting fell outside the intended scope, when the outcomes were exclusively clinical or physiological, or when the publication did not contribute directly to the review question or framework.

To reduce selective citation, recent high-level reviews were prioritized for mapping established evidence, original sources were prioritized for foundational theories and definitions, and representative PE-specific empirical studies were retained when they clarified proposed curriculum mechanisms. Backward and forward citation tracking was used to identify influential, contrasting, and frequently cited studies. Publications were compared across domains to avoid relying on multiple sources that made the same conceptual contribution while overlooking contradictory or boundary-defining evidence.

These procedures were intended to make the review boundaries and selection logic transparent rather than to imply exhaustive study identification or formal systematic-review screening. Consequently, no PRISMA flow diagram, protocol registration, pooled effect estimation, or study-level risk-of-bias assessment was undertaken.

### 2.4. Synthesis and Framework Development

The retained literature was synthesized comparatively across domains rather than treated as a homogeneous evidence base. No numerical weighting was assigned to individual domains because they contributed different forms of evidence and served different functions within the conceptual synthesis. The analysis examined: (1) where PE-specific empirical evidence was available; (2) where support came from adjacent physical activity, educational, psychological, or health-behavior research; (3) where different studies used overlapping terminology for related constructs; and (4) where the proposed relationships remained theory-informed conceptual propositions.

The framework was developed through an iterative process of concept comparison, domain integration, and refinement of curriculum-to-lifestyle pathways. Particular attention was given to relationships among curriculum design, teaching climate, embodied experience, motivation, self-efficacy, self-regulation, stress and emotion regulation, recovery-related awareness, and transfer beyond PE lessons.

Claims were calibrated according to the nature of the supporting evidence. Direct PE findings are described as associations or observed educational effects. Relationships supported mainly by adjacent evidence or theoretical reasoning are described as proposed pathways that may warrant empirical testing. The resulting framework should therefore be interpreted as a structured, evidence-informed conceptual model rather than as a validated causal model or an estimate of intervention effectiveness.

## 3. Existing Evidence and the Curriculum-to-Lifestyle Gap

A substantial review of the literature was performed, examining relationships among PE, physical activity, sport, physical literacy, and psychological or educational outcomes in adolescents and young adults. School-based reviews have considered depression, anxiety, self-esteem, self-efficacy, life satisfaction, general well-being, academic functioning, and broader health indicators (Bailey, 2006; Biddle & Asare, 2011; Fu et al., 2025; Poitras et al., 2016; Rocliffe et al., 2024; Rodriguez-Ayllon et al., 2019). Meta-analytic evidence further indicates that physical activity interventions may have broader educational relevance through selected cognitive outcomes. In adolescents and young adults, acute interventions were associated with improvements in processing speed, attention, and inhibitory control, whereas findings for chronic interventions and academic performance were less consistent (Haverkamp et al., 2020). In tertiary education, physical activity and physical literacy have also been associated with well-being, resilience, life satisfaction, and lower psychological distress (Leung et al., 2025). Collectively, this evidence supports the relevance of movement-related experiences to student well-being and educational functioning, but it is primarily organized around whether participation, exposure, fitness, or literacy-related constructs are associated with measurable outcomes.

A second body of scholarship has examined how PE is designed and experienced. Health-based PE, meaningful PE, pedagogical-model research, and physical-literacy scholarship emphasize curriculum design, motivation, competence, personal relevance, social interaction, and lifelong participation (Beni et al., 2017; Cairney et al., 2019; Dudley, 2015; Edwards et al., 2018; Fernández-Río et al., 2024; Haerens et al., 2011; Quennerstedt, 2019; Tremblay et al., 2018; Wintle, 2022). Health-literacy frameworks further emphasize the ability to access, understand, appraise, and apply health information in everyday life (Nutbeam, 2000; Sørensen et al., 2012). Research on Self-Determination Theory has shown the importance of autonomy, competence, relatedness, and need-supportive teaching for engagement and motivation (Ryan & Deci, 2000; Teixeira et al., 2012; Vasconcellos et al., 2020). In parallel, lifestyle research increasingly treats physical activity, sedentary behavior, sleep, diet, and recovery as interacting components of daily behavioral organization rather than as isolated exposures (Cruz et al., 2024; Dutil et al., 2022; Tremblay et al., 2016).

Despite these advances, a specific curriculum-level question remains insufficiently developed. Existing reviews mainly ask whether PE, physical activity, sport, or physical literacy is associated with mental health, health, or educational outcomes. They provide less explanation of how the PE curriculum, pedagogy, assessment, reflection, and peer interaction may help students interpret embodied experiences and develop transferable capacities for lifestyle self-regulation.

The present review addresses this gap by proposing a curriculum-to-lifestyle transfer framework. Its contribution is not to replace physical literacy, health literacy, meaningful PE, or health-based PE. Physical literacy provides a foundation in motivation, confidence, competence, knowledge, and lifelong participation; health literacy contributes processes of accessing, understanding, appraising, and applying health information; meaningful PE emphasizes personally relevant and engaging movement experiences; and health-based PE explains how health may be learned through embodied, social, and reflective practice.

The distinct contribution of the present framework is to connect these foundations with a broader sequence of curriculum processes and proposed transfer mechanisms. It links curriculum design, teaching climate, assessment, peer interaction, embodied experience, and reflection with motivation, self-efficacy, stress and emotion regulation, recovery-related awareness, and everyday lifestyle decision-making. It also specifies that transfer beyond PE is recursive, conditional, and influenced by peer, family, institutional, community, and cultural contexts.

The framework therefore extends established perspectives by focusing on how learning within PE may be translated into lifestyle self-regulation, while distinguishing evidence-supported educational processes from longer-term transfer pathways that remain to be tested.

Table 1 summarizes how the principal evidence domains contribute to this framework and where the remaining curriculum-to-lifestyle gap lies.

This synthesis provides the basis for the next section, which defines PE as a lifestyle learning environment and specifies the curriculum processes through which embodied learning may be connected with everyday self-regulation.

## 4. Physical Education as a Lifestyle Learning Environment

Reframing physical education as a lifestyle learning environment requires a shift in how PE is understood. In many educational and public health discussions, PE is valued because it offers structured opportunities for movement, contributes to physical activity accumulation, and supports motor skill development. These aims remain legitimate, but they do not fully capture the educational potential of PE. Quality PE is expected to support learning, inclusion, lifelong participation, health, and personal development, rather than only immediate activity time (UNESCO, 2015). Professional standards similarly emphasize that students should develop competence, confidence, knowledge, responsible participation, and the capacity to value physical activity across life contexts (SHAPE America, 2024). From this perspective, PE should not be reduced to a scheduled period in which students are made physically active. It is better understood as a structured educational setting in which students learn how bodily experience, motivation, health knowledge, social interaction, and daily routines are connected.

This distinction between activity delivery and lifestyle learning is central to the present review. Activity delivery focuses mainly on what students do during class: whether they move, how intensely they move, and whether activity-related recommendations are met. Lifestyle learning asks a different question: what students come to understand, value, regulate, and transfer from PE into everyday life. Earlier public health arguments for PE emphasized its contribution to population-level physical activity and health promotion (Sallis & McKenzie, 1991). Health-based PE scholarship, however, has argued that PE should move beyond narrow fitness testing and sport technique toward pedagogies that help students understand and enact health in personally meaningful ways (Fernández-Río, 2016; Haerens et al., 2011). Quennerstedt’s argument that students can learn health through PE further supports this view, because health is not treated as a fixed outcome delivered by teachers, but as something learned through embodied, social, and reflective experiences (Quennerstedt, 2019).

A lifestyle learning environment also depends on curriculum design. Pedagogical models in PE provide different ways of organizing learning tasks, student roles, assessment, responsibility, and teacher–student interaction (Fernández-Río et al., 2024). This matters because PE does not become educationally meaningful simply because students are active. Students may participate in PE while feeling excluded, anxious, bored, over-evaluated, or disconnected from their daily lives. Conversely, PE can become meaningful when students experience challenge, enjoyment, social connection, motor competence, personal relevance, and opportunities for reflection (Beni et al., 2017). Lifestyle-oriented PE perspectives similarly emphasize that activities should be experienced as personally meaningful and connected with forms of participation that students may sustain beyond formal schooling (Wintle, 2022). These features are important because lifestyle behavior is rarely sustained by information or external instruction alone. It is shaped by whether students experience movement as worthwhile, manageable, socially supported, and connected with their own identities and routines.

Empirical PE curriculum studies provide preliminary support for these proposed mechanisms. In a teacher-focused intervention, secondary-school PE teachers were trained to adopt more autonomy-supportive instructional practices. Students subsequently reported more adaptive psychological need satisfaction, autonomous motivation, and classroom engagement than students taught by teachers in the comparison condition (Cheon et al., 2012). In a Sport Education intervention, students taught through an extended season incorporating stable teams, student roles, and shared responsibility reported greater enjoyment and perceived effort than students receiving a more traditional instructional approach (Wallhead & Ntoumanis, 2004).

Responsibility-oriented curriculum models provide a further example. Studies implementing the Teaching Personal and Social Responsibility model within PE have reported improvements in selected dimensions of self-efficacy, self-regulated learning, and observed responsibility behaviors, although findings have been context- and sample-dependent (Escartí et al., 2010). Similarly, a health-based PE professional-development program in Hong Kong linked greater teacher participation with higher student-reported physical literacy, motivation, and enjoyment across follow-up assessments (Sum et al., 2022).

These studies do not demonstrate that curriculum changes automatically produce lasting lifestyle self-regulation. They do, however, support the more proximal proposition that teaching climate, curriculum organization, student responsibility, and reflective participation can shape how students experience motivation, competence, effort, and health-related learning within PE.

Physical literacy provides one foundation for this broader view of PE. Consensus definitions describe physical literacy as involving motivation, confidence, physical competence, knowledge, and understanding that support engagement in physical activity across the life course (Tremblay et al., 2018). Conceptual models similarly link physical literacy with health, physical activity, and broader developmental outcomes (Cairney et al., 2019). Additional international physical literacy statements reinforce that physical literacy should be understood as a lifelong and person-centered capability rather than as a narrow measure of fitness or motor proficiency (International Physical Literacy Association, n.d.). However, the present review does not treat physical literacy as the whole framework. Rather, physical literacy is one component of a wider lifestyle learning environment. A student may develop movement competence and confidence, yet still struggle to regulate stress, sleep, recovery, energy, emotion, or motivation across daily contexts. Measurement reviews also show that physical literacy is multidimensional and difficult to capture through simple indicators (Edwards et al., 2018). This reinforces the need to position PE within a broader learning model that includes, but extends beyond, physical competence and activity participation.

Health literacy offers another conceptual bridge. Health literacy has been defined as the ability to access, understand, appraise, and apply health-related information in daily life (Nutbeam, 2000; Sørensen et al., 2012). Applied to PE, this means that students should not only receive messages about being active or healthy. They should also learn how to interpret bodily cues, evaluate health information, make decisions about movement and recovery, and connect classroom experiences with daily routines. For example, an aerobic activity lesson can become a lifestyle learning experience when students are guided to notice effort, breathing, fatigue, mood, hydration, sleep, and recovery. A fitness unit can become more than performance testing when it helps students set realistic goals, monitor progress, reflect on barriers, and understand how activity choices fit into daily stress and energy management. In this sense, PE can serve as an embodied context for health literacy, not merely as a delivery channel for health information.

The proposed framework builds on these established perspectives but addresses a different level of explanation. Physical literacy primarily concerns the capacities and dispositions that support engagement in movement, whereas health literacy concerns the interpretation and use of health-related information. Meaningful PE focuses on the quality and personal significance of movement experiences, and health-based PE emphasizes how health can be learned through curriculum and pedagogy. The present framework integrates these contributions but places additional emphasis on curriculum-to-lifestyle transfer.

Specifically, it asks how movement experiences may be connected with self-monitoring, stress and emotion regulation, recovery-related awareness, and everyday decision-making beyond the lesson. It also includes feedback processes and ecological conditions that may reinforce, modify, or constrain transfer. The framework should therefore be understood as complementary and integrative rather than as a competing replacement for established PE and literacy models.

The concept of a lifestyle learning environment also requires attention to student agency. Adolescents and young adults are developing greater responsibility for their own routines, including physical activity, sedentary behavior, sleep, nutrition-related choices, social relationships, and academic demands. PE may support this transition when teaching practices provide meaningful choice, progressive challenge, reflective feedback, and opportunities for responsibility. PE-specific intervention studies suggest that these curriculum features can influence proximal outcomes such as need satisfaction, autonomous motivation, enjoyment, self-efficacy, and perceived physical literacy, although evidence for sustained transfer beyond PE remains limited (Cheon et al., 2012; Escartí et al., 2010; Sum et al., 2022; Wallhead & Ntoumanis, 2004). Self-Determination Theory suggests that learning environments supporting autonomy, competence, and relatedness are more likely to foster internalized motivation and adaptive engagement (Ryan & Deci, 2000). Self-efficacy theory similarly suggests that students’ beliefs about their capability influence effort, persistence, and behavior change (Bandura, 1977). Therefore, PE curriculum should not only ask whether students complete activities, but also whether they learn to make sense of their capabilities, regulate effort, and apply movement-related knowledge outside class.

These learning processes do not occur independently of students’ wider environments. From an ecological perspective, PE experiences are embedded within interacting classroom, peer, family, institutional, community, cultural, and policy contexts (Bronfenbrenner, 1979). A student may acquire goal-setting or recovery-related knowledge in PE but have limited opportunity to apply it because of family routines, academic demands, neighborhood safety, financial constraints, cultural expectations, or restricted access to appropriate facilities. Conversely, supportive peers, family encouragement, institutional resources, and accessible community opportunities may reinforce learning initiated in PE.

The framework should therefore not be interpreted as a universal curriculum prescription. PE structures differ across countries and educational systems in compulsory status, curriculum content, instructional time, assessment practices, teacher preparation, and links with health education. The proposed principles require contextual adaptation, and the effects of similar teaching strategies may vary according to the surrounding educational and social ecology.

This reframing may also be relevant in selected higher-education contexts, although university-level PE provision varies substantially across countries and institutions. In this review, “university PE” does not refer to a universally standardized degree program or to a direct continuation of school PE. Rather, it includes structured movement- or wellness-oriented educational provision for university students, such as compulsory or elective physical activity courses, general education activity modules, credit-bearing fitness or movement classes, and campus wellness courses. In some systems, comparable learning may instead be located within sport science, coaching, recreation, or health-promotion programs.

Students in higher education may experience irregular schedules, sedentary study demands, academic stress, reduced structured activity, and increasing responsibility for health behaviors, although these patterns vary across institutions and populations. Whole-system approaches to healthy universities emphasize that higher-education settings can shape health, learning, and well-being through coordinated environments, policies, curricula, and student support (Dooris et al., 2018). Where movement- or wellness-oriented courses exist, they may provide structured opportunities for young adults to reflect on movement, stress, recovery, and lifestyle self-management. This extension must remain context-sensitive and bounded. Such courses should not replace clinical care, nutrition counseling, or sleep medicine; rather, they may provide educational opportunities that connect movement experience with self-knowledge and practical regulation.

**Figure 1:**
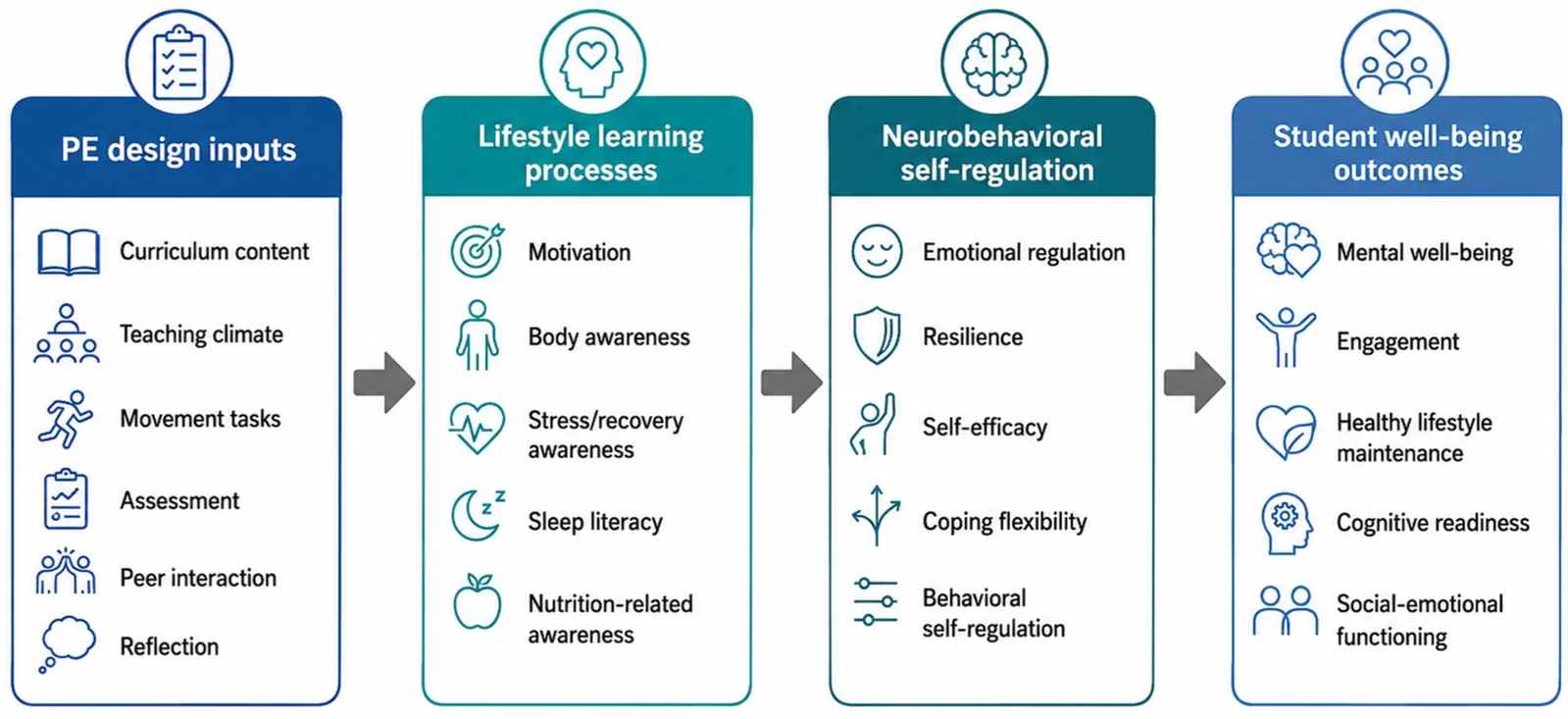
Physical education as a lifestyle learning environment for neurobehavioral well-being. PE design inputs are linked with lifestyle-learning processes and proposed neurobehavioral self-regulation outcomes. The pathways are recursive, context-dependent, and not causally established.

This understanding changes the educational question. The question is not only whether PE increases activity minutes or improves fitness scores, but whether it helps students develop transferable capacities for lifestyle self-regulation. These capacities include interpreting bodily signals, setting realistic movement goals, coping with stress, regulating emotions, understanding recovery, recognizing the role of sleep and nutrition-related behaviors, and sustaining motivation. In this sense, PE may contribute to neurobehavioral well-being by organizing repeated, embodied, and socially supported opportunities for students to practice regulation through movement. This does not imply that PE alone can solve students’ mental health or lifestyle challenges. Rather, it suggests that PE may occupy a distinctive educational position by connecting physical experience, health learning, motivation, and self-regulation within an embodied curriculum context. This conceptual logic is summarized in Figure 1, which integrates PE curriculum and pedagogy, physical literacy and health literacy, motivational processes, emotional regulation, self-efficacy, resilience, and lifestyle-related awareness into a curriculum-based framework (Bandura, 1977; Beni et al., 2017; Bronfenbrenner, 1979; Cairney et al., 2019; Cheon et al., 2012; Dooris et al., 2018; Edwards et al., 2018; Escartí et al., 2010; Fernández-Río, 2016; Fernández-Río et al., 2024; Gross, 2002; Haerens et al., 2011; International Physical Literacy Association, n.d.; Masten, 2001; Nutbeam, 2000; Quennerstedt, 2019; Ryan & Deci, 2000; Sallis & McKenzie, 1991; SHAPE America, 2024; Sørensen et al., 2012; Sum et al., 2022; Tremblay et al., 2018; UNESCO, 2015; Wallhead & Ntoumanis, 2004; Wintle, 2022). In this framework, “neurobehavioral self-regulation” refers to interacting motivational, emotional, cognitive-regulatory, and behavioral processes, rather than to directly measured neurological or neurobiological change.

For the purposes of this review, PE as a lifestyle learning environment refers to a curriculum-based context in which movement experiences, teaching climate, peer interaction, reflection, and health-related learning are intentionally aligned to support students’ lifestyle self-regulation and neurobehavioral well-being. This definition provides the conceptual foundation for the next section, which examines the neurobehavioral pathways through which PE may support motivation, stress regulation, emotional regulation, resilience, self-efficacy, and self-regulation.

## 5. Neurobehavioral Pathways: Motivation, Stress Regulation, Emotional Regulation, and Resilience

If PE is reframed as a lifestyle learning environment, its contribution to student well-being cannot be explained only by the amount of physical activity performed during class. A more adequate account requires attention to the pathways through which students experience movement, interpret bodily states, interact with peers and teachers, regulate emotions, and connect PE learning with daily life. In this review, the term “neurobehavioral pathways” refers to proposed links among educational conditions, psychological regulation, behavioral self-management, and embodied experience. Educational conditions include curriculum design, teaching climate, assessment, peer interaction, and reflective learning. Psychological processes include motivation, self-efficacy, stress appraisal, emotion regulation, and resilience. Behavioral processes include goal setting, self-monitoring, coping, effort regulation, activity planning, and recovery-related decision-making. The term does not denote direct neurobiological mechanisms. The review does not evaluate neural activation, brain structure or function, neurochemical responses, or biological markers, and it does not claim that ordinary PE lessons produce clinical neurological change. The proposed pathways are therefore primarily educational, psychological, and behavioral, although they concern the integrated regulation of cognition, emotion, behavior, and embodied experience.

Motivation is the first pathway because students’ experiences in PE can shape whether movement is perceived as meaningful, threatening, imposed, enjoyable, or personally relevant. Self-Determination Theory proposes that autonomy, competence, and relatedness are basic psychological needs that support motivation, development, and well-being (Ryan & Deci, 2000). The theory also explains why internalization matters for sustained behavior rather than short-term compliance alone (Deci & Ryan, 2000). In physical activity and PE research, more autonomous forms of motivation and need-supportive teaching have been associated with adaptive outcomes such as psychological need satisfaction, engagement, enjoyment, and behavioral persistence (Teixeira et al., 2012; Vasconcellos et al., 2020).

These findings do not demonstrate that need-supportive PE automatically produces long-term lifestyle change. Current evidence is stronger for proximal outcomes within PE than for sustained transfer into activity, recovery, sleep, or other lifestyle behaviors beyond the curriculum. Need-supportive teaching may create favorable motivational conditions, but transfer is likely to depend on additional processes such as repeated mastery experiences, explicit reflection, goal setting, action planning, access to activity opportunities, and reinforcement across social and environmental contexts.

The framework, therefore, treats autonomy support, competence support, and relatedness support as potentially important motivational mechanisms rather than as sufficient causes of long-term behavior change. Their value lies in increasing the likelihood that students experience movement as personally meaningful and manageable, while the durability and generalization of these effects require further longitudinal and context-sensitive research.

A second pathway involves stress regulation. Stress is not only a physiological response, but also a process of appraisal and coping. Classic stress and coping theory emphasizes that individuals evaluate demands, resources, and possible responses in stressful encounters (Folkman et al., 1986). PE may provide a setting in which students encounter manageable forms of challenge, including physical effort, unfamiliar skills, competition, cooperation, feedback, and social visibility. These experiences can be appraised either as a threat or as a challenge, depending on the teaching climate, peer context, and perceived competence. When PE is organized around progressive mastery, safety, and reflection, students may learn that effort, discomfort, and temporary failure can be interpreted and managed. When it is organized around humiliation, rigid comparison, or exclusion, the same activities may become stress-amplifying. This distinction is important because reviews of physical activity and mental health show promising but heterogeneous evidence, suggesting that the context and meaning of activity may matter as much as activity exposure itself (Biddle & Asare, 2011; Fu et al., 2025; Rocliffe et al., 2024; Rodriguez-Ayllon et al., 2019).

PE-specific intervention evidence provides preliminary support for this pathway when coping skills are taught explicitly. Lang et al. incorporated an experiential stress-management and coping program into regular PE classes for vocational-school students. Eight classes participated, with four allocated to the intervention condition and four to a control condition. Compared with the control group, students in the intervention group demonstrated improved adaptive coping skills from pre-intervention to post-intervention, followed by lower perceived stress at the six-month follow-up. Path analysis further suggested an indirect relationship between improved coping and later reductions in perceived stress (Lang et al., 2016).

This study suggests that PE may provide a feasible setting for structured coping instruction and experiential skill practice. However, it does not establish that PE participation, physical effort, or exposure to challenge alone automatically improves stress regulation. The evidence is more specific: when PE deliberately includes coping strategies, reflection, supported practice, and opportunities to interpret bodily and emotional responses, it may help students develop adaptive coping skills. The broader proposition that mastery-oriented or supportive PE alone produces transferable stress regulation remains theoretical and requires replication across different age groups, curricula, and cultural settings.

Movement experiences may also interact with stress beyond the PE classroom. Broader review evidence indicates that stress can reduce physical activity participation, while physical activity may be associated with stress-related processes (Stults-Kolehmainen & Sinha, 2014). For students, the educational relevance lies not simply in completing activity tasks, but in learning to recognize relationships among movement, breathing, fatigue, attention, mood, and coping. PE may provide an embodied context for such learning when these relationships are made explicit through guided reflection, coping practice, and supportive discussion. This does not require PE teachers to act as mental health clinicians. Rather, it requires clearly bounded pedagogical activities that help students notice bodily signals, reflect on effort and emotional responses, and consider how selected coping strategies might be applied in everyday situations. Whether these classroom experiences produce sustained stress-management behavior outside PE remains uncertain.

A third pathway involves emotional regulation. Emotion regulation refers to the processes through which individuals influence the emotions they have, when they have them, and how these emotions are experienced and expressed (Gross, 2002). PE is emotionally charged because it involves public performance, bodily exposure, peer comparison, success, failure, competition, cooperation, and feedback. These conditions may trigger anxiety, pride, shame, frustration, enjoyment, or belonging. Research on individual differences in emotion regulation also shows that strategies such as cognitive reappraisal and expressive suppression are associated with different affective and social outcomes (Gross & John, 2003). PE can therefore be understood as a setting in which students learn, implicitly or explicitly, how to respond to emotional experiences associated with bodily challenge and social evaluation.

PE-specific intervention evidence provides preliminary support for this pathway when emotional and interpersonal learning is embedded deliberately within curriculum design. Nazari et al. examined a hybrid Teaching Games for Understanding and Sport Education unit delivered to 72 school students over 16 sessions across two months. Compared with baseline, students demonstrated improvement in emotion regulation, together with gains in metacognition, intrapersonal intelligence, and psychomotor performance (Nazari et al., 2025).

These findings suggest that student-centered pedagogical models may create opportunities to practice emotional self-management within PE. Relevant features include shared decision-making, tactical problem solving, cooperation, role responsibility, and reflection. However, the study does not establish that conventional PE participation or physical exertion alone improves emotion regulation, and it does not demonstrate sustained transfer beyond the intervention period. The evidence should therefore be interpreted as support for a deliberately structured curriculum mechanism rather than for a general effect of PE.

Evidence from broader exercise research suggests possible relationships between movement and emotion regulation (Bernstein & McNally, 2017, 2018), but such findings cannot be transferred directly to PE curricula. Exercise exposure, curriculum design, teaching climate, peer interaction, and reflective learning are not equivalent conditions. In PE, the more defensible educational proposition is that repeated, structured opportunities to recognize, label, reflect on, and respond to emotions may support the practice of adaptive regulation during discomfort, frustration, competition, social comparison, and achievement. Whether these skills generalize to everyday emotional functioning remains uncertain and requires longitudinal and context-sensitive investigation. This perspective fits the broader thesis of PE as lifestyle learning: students are not only learning sports or exercises; they are also learning how to regulate themselves in embodied and social situations.

A fourth set of processes concerns self-efficacy, coping, resilience, and life skills. Although these constructs are related, they represent different aspects of adaptation and should not be treated as interchangeable.

Self-efficacy refers to an individual’s belief in their capability to organize and perform the actions required in a specific situation (Bandura, 1977). In PE, self-efficacy may be shaped by mastery experiences, feedback, social modeling, and the interpretation of bodily and emotional responses. A student who experiences achievable challenge and visible progress may become more confident in managing effort or attempting unfamiliar movement tasks. By contrast, repeated failure, public comparison, or exclusion may weaken perceived capability and contribute to avoidance. Self-efficacy is therefore best understood as a task- and context-specific belief rather than as a general indicator of resilience (Neumann et al., 2022).

Coping refers to the cognitive and behavioral strategies used to manage demands that are appraised as stressful or challenging (Folkman et al., 1986). Within PE, coping may involve regulating breathing, reframing temporary failure, seeking support, adjusting effort, or using problem-solving strategies during competition or skill learning. Coping is more situational and process-oriented than self-efficacy-based. Students may believe that they are capable of managing a challenge yet still use ineffective coping strategies, or they may develop adaptive coping strategies despite initially low confidence.

Resilience refers to the dynamic process through which individuals maintain, regain, or reorganize adaptive functioning in the presence of adversity (Masten, 2001; Southwick et al., 2014). It is broader than a single coping response and should not be inferred from short-term success in a PE task. In this framework, resilience is treated as a possible longer-term developmental outcome arising from repeated interactions among personal resources, supportive relationships, meaningful challenge, and environmental opportunities. Current PE evidence does not establish that participation alone produces resilience, and any such effect is likely to depend on curriculum quality, social context, and the accumulation of experiences over time.

Life skills are transferable competencies that may be taught and practiced explicitly, including goal setting, communication, cooperation, problem solving, emotional control, responsibility, and self-direction. Responsibility-based approaches such as Teaching Personal and Social Responsibility have been used in PE to structure opportunities for students to practice these competencies (Aygun et al., 2024; Pozo et al., 2018). Such programs may support selected social, emotional, and behavioral outcomes, but their effects should not be interpreted as equivalent to general resilience or as proof of transfer across all life contexts.

These constructs may interact, but they remain conceptually distinct. Self-efficacy may influence whether students attempt and persist with coping strategies. Coping may contribute to adaptive responses during challenge. Life-skills curricula may provide opportunities to practice goal setting, communication, and responsibility. Repeated successful adaptation may, over time, contribute to resilience. However, a change in one construct should not be assumed to indicate a change in the others. Future PE research should therefore measure self-efficacy, coping, resilience, and life skills separately and specify the proposed relationships among them.

Finally, these pathways converge in self-regulation. Self-regulated learning involves cyclical processes of goal setting, action, self-monitoring, feedback interpretation, strategy adjustment, and reflection (Zimmerman, 2002). In PE, these processes may include selecting an achievable movement goal, monitoring effort and emotional responses, evaluating progress, modifying a strategy, and deciding how the experience may be applied outside class.

This process is better understood as a feedback loop than as a one-directional transition. A successful mastery experience may strengthen self-efficacy, increase willingness to attempt future challenges, and create further opportunities for learning. Conversely, repeated failure, public comparison, or exclusion may weaken perceived capability and contribute to avoidance. Subsequent participation then generates new information about competence, effort, social acceptance, and behavioral consequences, which may reinforce or revise students’ beliefs and future choices (Bandura, 1977; Zimmerman, 2002).

Self-Determination Theory, self-efficacy theory, and self-regulated learning therefore serve related but distinct functions in the framework. Self-Determination Theory explains how autonomy-, competence-, and relatedness-supportive environments may facilitate motivation and internalization. Self-efficacy theory explains how beliefs about capability shape effort and persistence. Self-regulated learning explains how goals, monitoring, feedback, reflection, and strategy adaptation may organize change across repeated learning experiences (Bandura, 1977; Ryan & Deci, 2000; Zimmerman, 2002).

Broader behavior-change frameworks add complementary constructs, including capability, opportunity, motivation, intention, planning, and action control (Ajzen, 1991; Michie et al., 2011; Schwarzer et al., 2011). These constructs should not be transferred mechanically into PE or treated as proof that classroom learning will produce long-term behavior change. Rather, they help identify testable mechanisms through which reflective and contextually supported PE experiences may contribute to lifestyle self-regulation.

Transfer beyond PE requires explicit curriculum design rather than an assumption that students will generalize classroom experiences independently. A practical transfer cycle may involve movement experience, structured reflection, a personally relevant plan, an everyday trial, follow-up feedback, and strategy adjustment.

Brief reflective journals can help students record what they noticed about effort, mood, confidence, frustration, fatigue, or recovery during a lesson. Goal-setting and implementation-intention tasks can invite students to select a feasible movement, coping, or recovery-related action outside PE. Students may then anticipate barriers and specify when and where the action will be attempted. Follow-up assignments may include a short activity–mood–recovery log, a home-based movement experiment, or a subsequent class discussion focused on what was attempted, what contextual barriers arose, and how the strategy might be revised.

These activities should remain brief, inclusive, developmentally appropriate, and adaptable to local conditions. They should not require disclosure of sensitive personal information, assume equal access to resources, or be used as clinical screening or moral evaluation of students’ lifestyles. Their purpose is to support self-monitoring and adaptive decision-making, not to guarantee long-term behavior change.

This convergence also raises the issue of transfer. Life skills research in youth sport and physical activity contexts indicates that skills such as goal setting, self-direction, responsibility, emotional control, and social cooperation do not automatically transfer to other settings unless transfer is made explicit, practiced, and reflected upon. Motivation, stress regulation, emotional regulation, resilience, self-efficacy, and self-regulation should therefore not be treated as separate outcomes. They are interrelated processes through which PE may support neurobehavioral well-being when curriculum design intentionally connects movement experiences with everyday life.

Taken together, these pathways suggest that PE may contribute to student well-being when movement experiences are organized as meaningful, autonomy-supportive, emotionally safe, progressively challenging, and reflective. The strongest defensible claim from the current literature is not that PE automatically improves mental health, nor that ordinary PE lessons directly produce neurobiological change. Rather, PE may provide repeated educational opportunities for adolescents and young adults to practice embodied forms of motivation, coping, emotional regulation, resilience, self-efficacy, and self-regulation. This interpretation prepares the ground for the next section, which considers how sleep, recovery, and nutrition-related awareness can be incorporated as supportive lifestyle modules without displacing PE’s central educational purpose.

Table 2 translates the proposed pathways into principal curriculum components, learning processes, neurobehavioral targets, and feasible indicators. The table draws on prior work on quality PE, health-based PE pedagogy, physical literacy, health literacy, motivation, stress and emotion regulation, resilience, self-efficacy, self-regulated learning, behavior change, and responsibility-based PE (Ajzen, 1991; Bandura, 1977; Bean et al., 2018; Beni et al., 2017; Bernstein & McNally, 2017, 2018; Bronfenbrenner, 1979; Cairney et al., 2019; Cheon et al., 2012; Deci & Ryan, 2000; Dooris et al., 2018; Dudley, 2015; Dutil et al., 2022; Edwards et al., 2018; Escartí et al., 2010; Fernández-Río, 2016; Fernández-Río et al., 2024; Folkman et al., 1986; Gross, 2002; Gross & John, 2003; Haerens et al., 2011; International Physical Literacy Association, n.d.; Lang et al., 2016; Leung et al., 2025; Masten, 2001; Michie et al., 2011; Miller & Brickman, 2004; Nazari et al., 2025; Neumann et al., 2022; Nutbeam, 2000; Pozo et al., 2018; Quennerstedt, 2019; Rodriguez-Ayllon et al., 2019; Ryan & Deci, 2000; Sallis & McKenzie, 1991; Schwarzer et al., 2011; SHAPE America, 2024; Southwick et al., 2014; Sørensen et al., 2012; Stults-Kolehmainen & Sinha, 2014; Sum et al., 2022; Teixeira et al., 2012; Tremblay et al., 2018; Tremblay et al., 2016; UNESCO, 2015; Vasconcellos et al., 2020; Wallhead & Ntoumanis, 2004; Wintle, 2022; Zimmerman, 2002).

## 6. Sleep, Recovery, and Nutrition-Related Awareness as Supportive Lifestyle Modules

The previous section argued that PE may support neurobehavioral well-being through motivational, emotional, stress-regulatory, resilience-related, self-efficacy, and self-regulatory pathways. These pathways, however, do not operate in isolation from students’ daily routines. Motivation, emotional regulation, stress appraisal, and engagement with movement are also shaped by sleep, recovery, sedentary behavior, hydration, and nutrition-related choices. For this reason, a lifestyle learning environment in PE should include sleep, recovery, and nutrition-related awareness as supportive modules. These modules should not displace PE’s central focus on movement, learning, and pedagogy. Rather, they can help students understand why movement experiences are connected with energy, fatigue, mood, attention, effort, recovery, and everyday decision-making.

Sleep, recovery, and nutrition-related awareness should be incorporated into PE in a supportive and bounded manner. PE teachers can reasonably address general, developmentally appropriate relationships among movement, fatigue, recovery, sleep, hydration, energy, mood, and daily routines. They may guide students in recognizing bodily cues, reflecting on effort and recovery, evaluating general health information, and making feasible lifestyle decisions.

The educational boundary must remain explicit. PE should not substitute for specialist health education, individualized counseling, or clinical care. PE teachers should not diagnose or treat sleep disorders, prescribe individualized diets or supplements, manage eating disorders or nutritional deficiencies, assess clinical mental-health conditions, or provide medical rehabilitation. Persistent sleep disturbance, unexplained fatigue, recurrent pain, suspected disordered eating, or other clinically concerning signs should be addressed through institutional referral procedures and appropriately qualified health professionals.

Where interdisciplinary resources are available, PE teachers may collaborate with health educators, dietitians, nurses, counselors, psychologists, and medical staff. In such arrangements, PE provides an embodied and reflective learning context, whereas specialist professionals retain responsibility for individualized assessment, treatment, and advanced guidance.

Sleep is particularly relevant for adolescents and young adults because it is closely linked with learning, emotion, behavior, and daily functioning. Movement behavior guidelines already conceptualize physical activity, sedentary behavior, and sleep as interdependent components of youth health rather than as separate behavioral categories (Tremblay et al., 2016). Review evidence also indicates that sleep duration is associated with multiple health indicators in school-aged children and youth, including emotional and behavioral outcomes (Chaput et al., 2016). Consensus recommendations on sleep duration further indicate that sleep needs vary developmentally across adolescence and young adulthood (Hirshkowitz et al., 2015). However, sleep patterns and barriers to adequate recovery are not uniform across populations. Depending on cultural, educational, socioeconomic, family, and institutional contexts, relevant influences may include academic or employment schedules, commuting, screen use, family responsibilities, housing conditions, and social routines. Evidence on sleep timing also suggests that sleep should be understood not only in terms of duration, but also in relation to daily scheduling and health indicators among children and adolescents (Dutil et al., 2022). School start time research further illustrates how sleep, learning, and adolescent functioning are shaped by educational environments and daily routines (Wahlstrom & Owens, 2017). For PE, these findings do not mean that teachers should provide clinical sleep counseling. Instead, they suggest that PE can help students connect sleep with bodily readiness, perceived exertion, attention, mood, injury-prevention awareness, motivation, and recovery after effort.

Recovery-related awareness provides a practical bridge between movement and lifestyle learning. In many PE settings, recovery is treated as something that occurs after class or outside the curriculum. Yet students regularly experience fatigue, soreness, breathlessness, frustration, or changes in mood during and after movement tasks. These experiences can become learning opportunities when teachers help students interpret them constructively. For example, cool-down activities, reflection prompts, perceived exertion scales, recovery logs, or brief discussions of sleep and rest can help students recognize that adaptation is not only a matter of effort, but also of regulation. This is consistent with self-regulated learning theory, which emphasizes goal setting, monitoring, strategy adjustment, and reflection (Zimmerman, 2002). It also aligns with self-efficacy theory, because students who understand how to manage effort and recovery may become more confident in their ability to participate in physical activity without feeling overwhelmed or discouraged (Bandura, 1977).

Nutrition-related awareness should be handled with similar caution. The present review does not argue that PE should become a specialized nutrition education course. Rather, PE can provide embodied contexts in which students learn how energy, hydration, meal timing, fatigue, concentration, and recovery relate to movement experiences. Recent scoping review evidence suggests that physical activity and diet are both associated with sleep quality and duration in adolescents, supporting the idea that these lifestyle behaviors should be considered together (Cruz et al., 2024). School-based food and nutrition education interventions have also been reviewed in relation to adolescent food consumption, indicating that educational settings can influence nutrition-related knowledge and behaviors, although effects vary across program design and implementation (de Medeiros et al., 2022). An umbrella review of school-based healthy eating interventions similarly suggests that adolescent nutrition behaviors are shaped by school context, program components, and broader health-promoting environments (Samad et al., 2024). Additional review evidence on school-based healthy eating interventions indicates that nutrition-related outcomes depend on intervention content, implementation quality, and the extent to which educational messages are connected with students’ lived contexts (Chaudhary et al., 2020). School-based interventions that combine nutrition and physical activity components also reinforce the educational relevance of addressing movement and nutrition-related behaviors together, although such evidence should be translated cautiously into PE curriculum contexts (Adom et al., 2020). For PE, the implication is not that every lesson should include nutrition content, but that selected PE units can integrate nutrition-related awareness when it directly supports movement, recovery, and self-regulation.

These lifestyle modules are most useful when they are embedded in PE learning rather than added as disconnected health messages. A fitness lesson, for example, can ask students to compare how sleep, hydration, or meal timing may influence perceived exertion and concentration. A games-based unit can include reflection on fatigue, recovery between efforts, and emotional responses to competition. A personal fitness or wellness unit can help students set activity goals while also considering sleep routines, recovery needs, and realistic nutrition-related decisions. In each case, the learning target is not expert knowledge of physiology, dietetics, or sleep medicine. The target is lifestyle awareness: the ability to notice, interpret, and apply everyday information about the body in ways that support well-being.

This approach also helps avoid a common limitation in health education: presenting lifestyle behaviors as abstract recommendations detached from students’ lived experiences. Health literacy frameworks emphasize the ability to access, understand, appraise, and use health information in daily contexts (Nutbeam, 2000; Sørensen et al., 2012). PE is well positioned to make such information experiential. Students can feel the effects of effort, notice changes in mood and attention, experience the consequences of poor preparation, and reflect on how daily routines shape movement quality. When combined with autonomy-supportive teaching, these experiences may support more internalized motivation and more realistic self-regulation (Deci & Ryan, 2000; Ryan & Deci, 2000; Teixeira et al., 2012; Vasconcellos et al., 2020). When combined with emotionally safe and meaningful pedagogy, they may also reduce the risk that lifestyle messages become moralizing, body-shaming, or overly performance-oriented (Beni et al., 2017; Fernández-Río, 2016; Fernández-Río et al., 2024; Haerens et al., 2011; Quennerstedt, 2019; Wintle, 2022).

**Figure 2:**
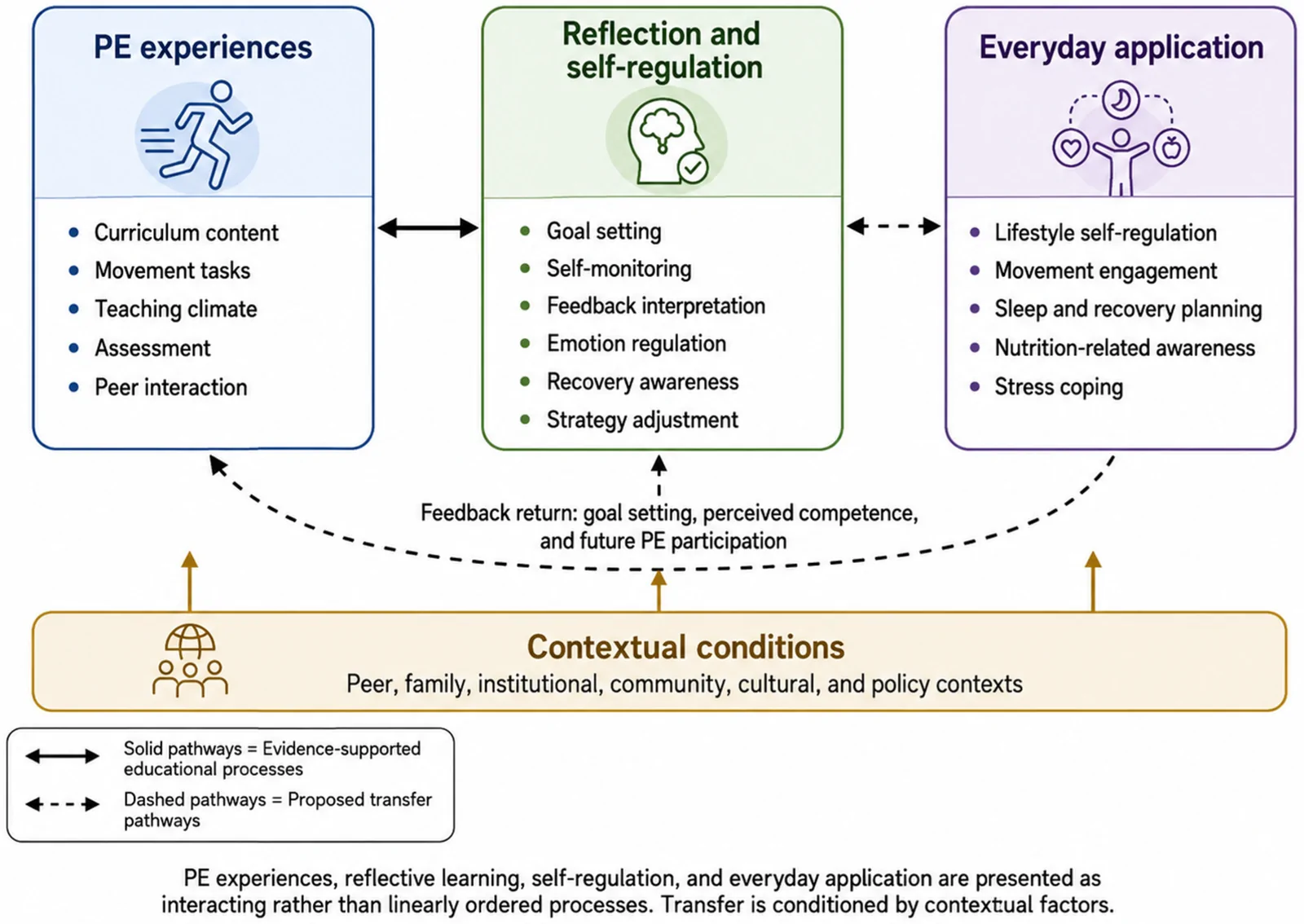
Recursive curriculum-to-lifestyle processes in physical education. PE experiences, reflection, self-regulation, and everyday application interact through feedback and are conditioned by wider social and institutional contexts. Solid pathways indicate established educational foundations; dashed pathways indicate proposed transfer processes requiring empirical testing.

Nevertheless, the inclusion of sleep, recovery, and nutrition-related awareness must remain developmentally appropriate and educationally bounded. PE teachers should not be expected to diagnose sleep problems, prescribe diets, or manage clinical mental health conditions. Instead, PE can help students develop foundational awareness of how lifestyle behaviors interact with movement and well-being. For adolescents, this may involve learning how sleep, screen time, meals, hydration, and activity influence school-day energy and mood. For young adults, particularly those in university settings, PE or wellness-oriented courses may also support reflection on stress, irregular routines, sedentary study patterns, and independent lifestyle decisions. In both groups, the potential educational value of PE lies in connecting movement experience with self-knowledge and practical regulation. Figure 2 summarizes this proposed curriculum-to-lifestyle process as recursive rather than linear. PE experiences may generate cognitive, emotional, behavioral, and social feedback; students may interpret this feedback through motivation, self-efficacy, prior experience, and perceived social acceptance; and teachers may adapt subsequent tasks, support, feedback, or reflection accordingly.

The components of the model differ in evidential status. Curriculum design, teaching climate, meaningful choice, goal setting, feedback, reflective learning, and proximal outcomes such as motivation, perceived competence, enjoyment, engagement, and selected self-regulatory skills have established foundations in PE and educational research. By contrast, the longer-term transfer of these classroom processes into sustained movement behavior, stress coping, sleep and recovery planning, nutrition-related decision-making, and broader lifestyle self-regulation remains conceptually proposed and less directly supported.

Transfer is also conditioned by opportunities and constraints within peer, family, institutional, community, cultural, and policy contexts. Figure 2 should therefore be interpreted as an evidence-informed conceptual model identifying testable relationships, not as a validated sequence in which PE participation necessarily produces long-term lifestyle change.

Thus, sleep, recovery, and nutrition-related awareness should be viewed as supportive lifestyle modules within PE, not as competing curriculum domains. They strengthen the broader argument that PE can contribute to neurobehavioral well-being when it helps students connect bodily experience, motivation, emotion, stress, recovery, and daily routines. This prepares the foundation for the next section, which integrates these elements into curriculum implications and future research directions.

## 7. Curriculum Implications and Priorities for Empirical Testing

The preceding sections proposed that PE may function as a lifestyle learning environment when movement tasks, teaching climate, reflection, and health-related learning are intentionally aligned (Bandura, 1977; Gross, 2002; Haerens et al., 2011; Masten, 2001; Nutbeam, 2000; Quennerstedt, 2019; Ryan & Deci, 2000; Sørensen et al., 2012). This does not require PE to abandon movement competence, motor learning, physical activity participation, or fitness. Rather, it requires these established curriculum goals to be taught in ways that also help students interpret effort, respond to challenge, regulate participation, and connect movement experiences with everyday routines (Bandura, 1977; Deci & Ryan, 2000; Fernández-Río et al., 2024; Haerens et al., 2011; Nutbeam, 2000; Pozo et al., 2018; Quennerstedt, 2019; Ryan & Deci, 2000; Sørensen et al., 2012; Teixeira et al., 2012; Vasconcellos et al., 2020; Zimmerman, 2002; Aygun et al., 2024).

The practical implications can be organized across three PE-specific levels: curriculum design, teaching enactment, and assessment. Curriculum design concerns what learning objectives are included within PE units and how lessons are sequenced. Teaching enactment concerns how movement tasks, interaction, feedback, choice, and reflection are organized during lessons (Aygun et al., 2024; Deci & Ryan, 2000; Fernández-Río et al., 2024; Pozo et al., 2018; Ryan & Deci, 2000; Teixeira et al., 2012; Vasconcellos et al., 2020). Assessment concerns what evidence of learning is collected and how that evidence is used to support further movement and lifestyle learning, including proximal processes such as goal setting, effort interpretation, strategy adjustment, self-monitoring, and reflection (Bandura, 1977; Fernández-Río et al., 2024; Haerens et al., 2011; Quennerstedt, 2019; Zimmerman, 2002).

### 7.1. PE Curriculum Design

At the curriculum level, lifestyle learning should be embedded within movement units rather than delivered as a detached health-information component. Health-based and embodied approaches to PE emphasize that health-related learning becomes educationally meaningful when it is connected with movement experience, curriculum design, reflection, and context rather than presented only as decontextualized information (Haerens et al., 2011; Nutbeam, 2000; Quennerstedt, 2019; Sørensen et al., 2012). Pedagogical-model and responsibility-based PE research further suggests that curriculum structure, task design, and implementation quality influence how students experience and interpret PE (Aygun et al., 2024; Fernández-Río et al., 2024; Pozo et al., 2018).

A fitness unit, for example, may combine aerobic or resistance activities with learning objectives related to pacing, effort interpretation, recovery, and realistic goal setting. A games unit may include objectives related to emotional responses, communication, cooperation, and decision-making under pressure. Outdoor, dance, or individual-activity units may create opportunities to examine confidence, personal preference, self-directed participation, and forms of movement that students may be willing to continue beyond formal PE.

Unit planning should identify a limited number of proximal learning objectives rather than attempt to address every lifestyle domain simultaneously. These objectives may include interpreting perceived exertion, selecting an achievable challenge, responding constructively to feedback, applying a coping strategy, cooperating within a group, or explaining how rest and recovery influence participation. Lifestyle-related content should remain directly connected to the movement experience of the lesson and should not displace motor learning, activity participation, physical competence, or meaningful engagement.

Curriculum sequencing is also important. A unit may begin with teacher-guided recognition of effort, emotional responses, and bodily cues; progress toward student goal setting, self-monitoring, and strategy selection; and conclude with an opportunity to test or reflect on a feasible application outside PE. This sequence makes transfer an explicit curriculum objective rather than an assumed consequence of participation. However, curriculum designers should avoid treating lifestyle learning as a uniform package. The relevance and feasibility of particular objectives will vary according to student age, developmental level, curriculum time, local policy, teacher preparation, resources, and cultural context.

### 7.2. Teaching Practice

At the lesson level, PE teachers can support lifestyle learning through concrete pedagogical choices. Autonomy-supportive strategies may include offering meaningful choices, explaining the purpose of learning tasks, acknowledging student difficulties, and reducing unnecessary public comparison (Deci & Ryan, 2000; Ryan & Deci, 2000; Teixeira et al., 2012; Vasconcellos et al., 2020). Competence-supportive strategies may include progressive challenge, differentiated tasks, mastery-oriented feedback, and opportunities for students to recognize progress without reducing self-worth to performance scores. Relatedness-supportive strategies may include cooperative learning, peer encouragement, inclusive grouping, shared responsibility, and emotionally safe classroom climates (Aygun et al., 2024; Deci & Ryan, 2000; Pozo et al., 2018; Ryan & Deci, 2000; Teixeira et al., 2012; Vasconcellos et al., 2020).

Meaningful choice does not require an unstructured lesson. Teachers may allow students to select among tasks that address the same learning objective at different levels of difficulty, choose a preferred role within a cooperative activity, or select a realistic personal goal within the boundaries of the unit. Progressive challenge may be created by adjusting space, equipment, rules, duration, intensity, or task complexity so that students experience attainable improvement. Mastery-oriented feedback should focus on strategy, effort regulation, task understanding, and individual progress rather than fixed judgments of ability or public comparison.

Reflection should remain brief and connected to action. Teachers might ask students to identify when effort became difficult, which strategy helped them persist, how peer interaction affected participation, or what change they would make in the next attempt. Short effort, emotion, and recovery check-ins can occur before or after activity without substantially reducing movement time. Cooperative tasks, rotating roles, peer feedback, and inclusive grouping may also provide opportunities to practice communication, responsibility, emotional regulation, and social problem solving within authentic PE activities (Aygun et al., 2024; Pozo et al., 2018).

Transfer-oriented teaching may include a short reflective journal, a goal-setting task, a movement experiment, or a follow-up question outside class. A subsequent lesson should then provide an opportunity to review what was attempted, identify barriers, interpret the experience, and revise the strategy. Life-skills research indicates that competencies learned in sport or physical activity settings do not automatically generalize to other life contexts unless transfer is explicitly discussed, practiced, and reflected upon (Bean et al., 2018). Positive youth-development research similarly suggests that social, emotional, and behavioral outcomes depend on program structure, relational climate, and intentional learning processes rather than participation alone (Holt et al., 2017).

These practices position PE teachers as facilitators of movement learning and reflective self-regulation rather than as clinicians or lifestyle counselors. Sleep, recovery, hydration, and nutrition-related awareness should therefore remain general, developmentally appropriate, and directly connected to participation and learning. Individualized diagnosis, treatment, dietary prescription, or management of clinically concerning problems should remain the responsibility of appropriately qualified health professionals.

### 7.3. Assessment for Lifestyle Learning

Assessment within this framework should extend beyond attendance, activity time, fitness scores, and sport-skill performance, while retaining these indicators where they are relevant to curriculum objectives. Health-based and pedagogical approaches to PE support assessment practices that capture learning, reflection, decision-making, and students’ interpretation of movement experiences rather than reducing achievement to performance outcomes alone (Fernández-Río et al., 2024; Haerens et al., 2011; Nutbeam, 2000; Quennerstedt, 2019; Sørensen et al., 2012).

Assessment for lifestyle learning should focus primarily on proximal learning processes that arise within PE. These may include the ability to set an achievable movement goal, interpret effort, use feedback, adjust a strategy, cooperate with peers, identify an emotional or coping response, or explain a basic recovery decision. Feasible formative approaches may include brief goal records, one- or two-item reflection prompts, student self-assessment, teacher observation of strategy use, peer feedback, and short movement–reflection tasks. For example, students may be asked to identify the strategy they used when a task became difficult, compare perceived effort before and after a pacing adjustment, or explain one realistic way in which the lesson might inform an activity decision outside class.

Assessment should be aligned with the specific construct and mechanism being examined. Motivation, perceived stress, emotion regulation, resilience, self-efficacy, engagement, recovery-related awareness, and lifestyle self-regulation are related but distinct and should not be combined into a generic outcome battery. Measure selection should therefore correspond to the curriculum component, the hypothesized mechanism, the population and setting, the expected time frame of change, and whether the intended outcome is proximal or distal.

Motivation measures should correspond to the framework’s need-supportive and internalization pathways. In school PE, instruments such as the Perceived Locus of Causality Questionnaire can distinguish autonomous, controlled, and amotivated forms of regulation toward participation in PE. Such measures are appropriate when the curriculum component involves meaningful choice, competence-supportive feedback, task relevance, or motivational climate (Deci & Ryan, 2000; Lonsdale et al., 2011; Ryan & Deci, 2000; Teixeira et al., 2012; Vasconcellos et al., 2020). Motivation scores should not be treated as direct indicators of stress regulation, resilience, or long-term behavior transfer. PE-specific versions of the Perceived Locus of Causality Questionnaire have been used to assess different forms of motivational regulation in school PE.

Perceived stress measures correspond to the framework’s stress-appraisal pathway. The Perceived Stress Scale assesses the extent to which individuals experience demands as unpredictable, uncontrollable, or overwhelming and may therefore be appropriate when an intervention is expected to influence general stress appraisal (Cohen et al., 1983). However, it does not measure coping-strategy use, physiological stress responses, or resilience directly. Where the curriculum specifically teaches coping strategies, stress appraisal should be supplemented by measures or qualitative indicators of strategy selection and use.

Emotion-regulation measures should be used when PE lessons explicitly target the recognition, interpretation, or regulation of emotional responses. Instruments such as the Emotion Regulation Questionnaire for Children and Adolescents (ERQ-CA) may assess habitual use of cognitive reappraisal and expressive suppression (Gullone & Taffe, 2012). These dimensions correspond to selected emotion-regulation strategies rather than to emotional well-being as a whole. Other measures may be required when the intervention targets emotional awareness, regulation difficulties, coping flexibility, or context-specific responses during PE. The ERQ-CA assesses emotion-regulation strategies in children and adolescents.

Resilience measures correspond to the framework’s proposed adaptive-functioning pathway. The Connor–Davidson Resilience Scale may be used when the research question concerns perceived capacity to adapt in the presence of stress or adversity (Connor & Davidson, 2003). Resilience scores should not be interpreted as direct evidence that students have acquired specific coping skills or transferred PE learning into everyday behavior. Because resilience is dynamic and context-dependent, scale scores may be strengthened by longitudinal assessment, student narratives, or evidence of adaptation across repeated challenges.

Self-efficacy measures should be task- and context-specific. They may assess confidence in performing a movement task, regulating effort, coping with challenge, planning activity, or continuing participation beyond PE, depending on the proposed mechanism (Bandura, 1977). A general confidence measure would not adequately capture these distinctions. Researchers should therefore specify the behavior, context, and time frame to which the self-efficacy judgment refers.

Recovery-related awareness measures should correspond to the supportive lifestyle modules proposed in the framework. General mental well-being or resilience scales cannot establish whether students understand recovery. More appropriate indicators may include recognition of fatigue and recovery cues; understanding of relationships among effort, rest, hydration, sleep, and readiness; and the ability to formulate a feasible recovery response. Because a single widely established PE-specific recovery-related awareness scale is not currently central to this framework, researchers may use brief curriculum-aligned knowledge items, scenario-based questions, reflective prompts, or structured observations. Any newly developed items should undergo content-validity, developmental-appropriateness, reliability, and construct-validity testing before being treated as established measures.

Lifestyle self-regulation measures should map onto the recursive processes of goal setting, planning, action, self-monitoring, feedback interpretation, reflection, and strategy adjustment (Ajzen, 1991; Michie et al., 2011; Schwarzer et al., 2011; Zimmerman, 2002). Depending on the study aim, researchers may use validated self-regulation questionnaires, goal records, planning measures, reflective journals, behavioral logs, or structured interviews. General self-regulation instruments can assess broad regulatory capacity, but curriculum-aligned measures are needed to determine whether students can apply a specific PE-based strategy. Self-regulation questionnaires assess processes involved in developing, implementing, monitoring, and adjusting goal-directed behavior, although their applicability should be evaluated for the relevant population and context.

Student-engagement measures correspond to students’ behavioral participation, emotional involvement, and cognitive investment in PE learning (Fredricks et al., 2004). Engagement should remain distinct from motivation: motivation concerns the reasons and regulatory basis for participation, whereas engagement concerns how students participate and invest effort during learning. In higher education, engagement may also reflect interactions among students, teachers, curriculum, and institutional context (Kahu, 2013; Kahu & Nelson, 2018). Higher-education engagement measures may therefore be useful when PE-based lifestyle learning is studied in university or wellness-oriented courses (Zhoc et al., 2019).

Mental well-being and flourishing measures should be treated primarily as broader or distal outcomes rather than direct measures of the curriculum mechanisms shown in the framework. The Warwick–Edinburgh Mental Well-being Scale may capture positive mental functioning, including validation work with teenage school students (Clarke et al., 2011; Tennant et al., 2007). The PERMA-Profiler and flourishing-related indicators may similarly assess broader positive psychological functioning (Butler & Kern, 2016; Diener et al., 2010). Changes in these outcomes may be educationally important, but they do not identify whether motivation, coping, emotion regulation, recovery-related awareness, or self-regulation produced the change.

Student well-being reviews further emphasize that well-being is multidimensional and influenced by personal, relational, educational, and institutional conditions (Hossain et al., 2023). Recent scoping-review evidence also highlights the roles of student voice, school environments, and educational conditions in promoting well-being (Murray et al., 2024). University student well-being is likewise shaped by psychological, social, institutional, and lifestyle-related factors (Gilmore et al., 2025). Broad well-being outcomes should therefore be interpreted alongside mechanism-specific measures and contextual information rather than attributed solely to a PE curriculum.

Candidate instruments are provided as examples rather than mandatory tools. Researchers should select measures according to the study question and verify their linguistic, developmental, and cultural validity in the population under investigation. Greater consistency in construct definitions and measurement may improve comparability across studies and facilitate future evidence synthesis. However, instrument standardization should not take priority over construct validity, cultural appropriateness, developmental suitability, or feasibility within the specific PE setting.

Assessment practices must also respect ethical and equity boundaries. Students should not be rewarded or penalized for body weight, appearance, diagnosed health conditions, sleep duration, dietary intake, or disclosure of sensitive personal information. Lifestyle behavior outside school should not be graded as though all students have equal access to time, facilities, family support, safe environments, or extracurricular opportunities. The purpose of assessment is to support learning, feedback, and strategy development rather than to judge the moral quality of students’ lifestyles.

### 7.4. Priority Directions for PE Research

Four priorities appear most urgent. First, mechanism-focused classroom studies should test how specific PE curriculum and teaching features influence proximal learning processes. Second, longitudinal and mixed-method studies should examine whether explicitly taught strategies are used beyond PE. Third, assessment studies should identify feasible and contextually valid indicators of PE-based lifestyle learning. Fourth, implementation studies should determine whether teachers can deliver these components with acceptable fidelity, burden, adaptability, and sustainability across different PE systems.

A first priority is to examine PE curriculum mechanisms. Studies should compare clearly specified units rather than broadly comparing PE participation with non-participation. Researchers might examine units with and without meaningful choice, structured reflection, mastery-oriented feedback, responsibility-based teaching, recovery-related awareness, or explicit transfer tasks. Outcomes should correspond to the proposed curriculum mechanism. For example, autonomy-supportive teaching should primarily be examined in relation to motivation and need satisfaction, while reflective goal-setting tasks may be examined in relation to self-monitoring, planning, and strategy adjustment. Pedagogical-model reviews and responsibility-based PE research suggest that curriculum structure and implementation quality influence educational outcomes (Aygun et al., 2024; Fernández-Río et al., 2024; Pozo et al., 2018).

Future intervention studies may also use complementary behavior-change frameworks to specify mechanisms more precisely. The Health Action Process Approach may help distinguish motivational processes, such as outcome expectations and self-efficacy, from volitional processes, such as planning, action control, and recovery from lapses. Similarly, the Behavior Change Wheel and its capability–opportunity–motivation framework may help researchers identify whether an intervention primarily targets student capability, social or environmental opportunity, motivation, or a combination of these mechanisms (Michie et al., 2011; Schwarzer et al., 2011).

These frameworks are proposed as design and analytic tools rather than as additional foundations that all PE curricula must adopt. Their value lies in making intervention components and hypothesized mechanisms explicit. For example, meaningful choice may primarily target autonomous motivation, a movement journal may target self-monitoring and action control, and access to extracurricular opportunities may address environmental opportunity. Such distinctions would allow future studies to examine why an intervention succeeds, for whom it works, and under which educational conditions.

A second priority is transfer beyond PE. Life-skills research indicates that skills learned in sport or physical activity settings do not automatically transfer to other life contexts unless transfer is explicitly discussed, practiced, and reflected upon (Bean et al., 2018). Positive youth-development research similarly suggests that social, emotional, and behavioral outcomes depend on program structure, relational climate, and intentional learning processes rather than participation alone (Holt et al., 2017). Longitudinal and mixed-method studies should therefore examine whether students use PE-based strategies in daily movement, stress coping, recovery, planning, or other lifestyle decisions. Such studies should distinguish stated intentions from enacted behavior and assess whether changes persist after a PE unit or course has ended.

Transfer studies should also examine contextual conditions. Family support, school schedules, university demands, access to facilities, transportation, community safety, cultural expectations, and socioeconomic resources may enable or constrain the use of PE-based learning outside class. Structural approaches to health-promoting learning environments indicate that student mental health and well-being are influenced by institutional design, educational environments, and coordinated support rather than by isolated individual-level strategies alone (Dooris et al., 2018; International Conference on Health Promoting Universities and Colleges, 2015; Olsson et al., 2024).

A third priority is measurement. Researchers should select indicators that correspond directly to the construct and mechanism being tested rather than administer broad batteries of unrelated psychological outcomes. Short measures, structured observations, student work samples, goal records, and mixed-method designs may be more feasible than extensive questionnaires in typical PE settings. Research should also determine whether proposed measures are developmentally appropriate, culturally valid, sensitive to change during a PE unit, and practical without excessive disruption of lesson time.

A fourth priority is implementation within school and university systems. Implementation research should document how teachers enact the curriculum, including task differentiation, feedback content, student choice, grouping practices, reflection time, assessment procedures, and adaptations made during lessons. Research on school-based physical activity interventions indicates that adoption, fidelity, reach, scalability, and maintenance should be considered when moving from promising designs to routine educational practice (Kennedy et al., 2021). Future PE studies should therefore include process evaluation examining not only whether outcomes changed, but also what was delivered, how students experienced it, which adaptations occurred, and what contextual conditions influenced implementation.

Whole-school and whole-community models emphasize that student health and learning are shaped by coordinated environments, policies, relationships, and services (Lewallen et al., 2015). More recent applications of whole-school health frameworks to mental-health support similarly suggest that student well-being requires coordinated systems rather than isolated classroom strategies (Lever et al., 2024). In higher education, health-promoting university frameworks and charters emphasize the influence of institutional culture, learning environments, policy, and community conditions (Dooris et al., 2018; International Conference on Health Promoting Universities and Colleges, 2015; Olsson et al., 2024). PE should not carry the entire responsibility for student well-being. Its distinctive contribution lies in providing an embodied curriculum space in which movement, feedback, social interaction, bodily awareness, and lifestyle learning can be integrated.

Table 3 prioritizes four research areas that are both theoretically important and feasible for PE-based studies: proximal classroom mechanisms, stress–emotion–recovery learning, transfer beyond PE, and implementation feasibility. The suggested indicators emphasize brief construct-specific measures, curriculum-aligned student work, structured observation, and mixed-method follow-up rather than an extensive generic outcome battery (Adom et al., 2020; Ajzen, 1991; Aygun et al., 2024; Bandura, 1977; Bean et al., 2018; Bernstein & McNally, 2017, 2018; Butler & Kern, 2016; Chaput et al., 2016; Chaudhary et al., 2020; Clarke et al., 2011; Cohen et al., 1983; Connor & Davidson, 2003; Cruz et al., 2024; Deci & Ryan, 2000; de Medeiros et al., 2022; Diener et al., 2010; Dooris et al., 2018; Fernández-Río et al., 2024; Folkman et al., 1986; Fredricks et al., 2004; Gilmore et al., 2025; Gross, 2002; Gross & John, 2003; Gullone & Taffe, 2012; Haerens et al., 2011; Hirshkowitz et al., 2015; Holt et al., 2017; Hossain et al., 2023; International Conference on Health Promoting Universities and Colleges, 2015; Kahu, 2013; Kahu & Nelson, 2018; Kennedy et al., 2021; Lang et al., 2016; Lever et al., 2024; Lewallen et al., 2015; Lonsdale et al., 2011; Masten, 2001; Michie et al., 2011; Miller & Brickman, 2004; Murray et al., 2024; Nazari et al., 2025; Neumann et al., 2022; Nutbeam, 2000; Olsson et al., 2024; Pozo et al., 2018; Quennerstedt, 2019; Ryan & Deci, 2000; Samad et al., 2024; Schwarzer et al., 2011; Sørensen et al., 2012; Southwick et al., 2014; Stults-Kolehmainen & Sinha, 2014; Teixeira et al., 2012; Tennant et al., 2007; Tremblay et al., 2016; Vasconcellos et al., 2020; Wahlstrom & Owens, 2017; Zhoc et al., 2019; Zimmerman, 2002).

Overall, PE may be understood as both education for movement and education through movement within the proposed framework. It does not imply that PE alone can prevent psychological distress or resolve complex lifestyle problems. Rather, PE may provide recurring and developmentally meaningful opportunities for students to practice movement-related self-regulation, reflection, and transfer within a clearly bounded educational setting.

## 8. Conclusions

The principal contribution of this narrative review is an evidence-informed, curriculum-oriented conceptual framework linking PE curriculum and pedagogy with proposed processes of lifestyle self-regulation in adolescents and young adults. Embodied movement experiences, teaching climate, reflection, motivation, self-efficacy, stress and emotion regulation, and recovery-related awareness are conceptualized as potentially interacting educational processes rather than as isolated outcomes.

The framework is not a standardized or empirically validated curriculum model, and it does not imply that PE alone can prevent mental distress, produce direct neurobiological change, or replace clinical, nutritional, or sleep-related care. Its educational mechanisms and transfer pathways remain context-dependent and require empirical testing across diverse school, university, cultural, and institutional settings.

Future research should prioritize mechanism-focused and longitudinal testing of clearly specified PE curricula, supported by implementation research across contrasting educational contexts and feasible mixed-methods assessment of student self-regulation and well-being. PE may therefore be understood as both education for movement and education through movement, while retaining fitness and skill development as essential components of its broader educational purpose.

## Figures and Tables

**Table 1:** Existing evidence domains and the remaining curriculum-to-lifestyle gap.

Evidence Domain	Main Contribution	Remaining Gap	Role in the Present Review
PE, physical activity, sport, and mental health	Links participation with psychological and educational outcomes (Bailey, 2006; Biddle & Asare, 2011; Fu et al., 2025; Poitras et al., 2016; Rocliffe et al., 2024; Rodriguez-Ayllon et al., 2019)	Curriculum mechanisms remain unclear	Shifts attention from exposure to learning
Tertiary physical activity and physical literacy	Links movement-related capacities with student well-being (Leung et al., 2025)	Young adults are rarely studied as learners	Extends the framework to higher education
PE curriculum, health-based PE, and meaningful experience	Explains how pedagogy shapes meaningful learning (Beni et al., 2017; Fernández-Río et al., 2024; Haerens et al., 2011; Quennerstedt, 2019; Wintle, 2022)	Lifestyle transfer is rarely specified	Connects embodied learning with regulation
Physical literacy and health literacy	Provides foundations for movement and health-related decision-making (Cairney et al., 2019; Dudley, 2015; Edwards et al., 2018; Nutbeam, 2000; Sørensen et al., 2012; Tremblay et al., 2018)	Curriculum-to-lifestyle processes remain underdeveloped	Supplies foundational capabilities
Motivation and need-supportive teaching	Explains how teaching climate shapes motivation and engagement (Ryan & Deci, 2000; Teixeira et al., 2012; Vasconcellos et al., 2020)	Motivation is often separated from transfer	Positions motivation as a mechanism
Sleep, recovery, nutrition, and movement behaviors	Situates behavior within interconnected daily routines (Cruz et al., 2024; Dutil et al., 2022; Tremblay et al., 2016)	These domains are rarely integrated into PE curriculum	Supports bounded lifestyle awareness

**Table 2:** Physical education curriculum components, neurobehavioral learning targets, and supporting literature.

PE Curriculum Component	Teaching Strategy	Lifestyle-Learning Process	Primary Target	Feasible Indicators	Key Supporting Literature
Movement skill and fitness learning	Progressive challenge and mastery feedback	Effort interpretation	Task-specific self-efficacy	Perceived competence; persistence	(Beni et al., 2017; Cairney et al., 2019; Dudley, 2015; Edwards et al., 2018; Fernández-Río, 2016; Fernández-Río et al., 2024; Haerens et al., 2011; Quennerstedt, 2019; SHAPE America, 2024; UNESCO, 2015)
Autonomy-supportive instruction	Meaningful choice and rationale	Internalization	Autonomous motivation	Motivation; class engagement	(Deci & Ryan, 2000; Miller & Brickman, 2004; Ryan & Deci, 2000; Teixeira et al., 2012; Vasconcellos et al., 2020)
Reflective movement tasks	Brief journals and goal reflection	Self-monitoring	Behavioral self-regulation	Goal record; strategy adjustment	(Folkman et al., 1986; Michie et al., 2011; Nutbeam, 2000; Schwarzer et al., 2011; Stults-Kolehmainen & Sinha, 2014; Sørensen et al., 2012; Zimmerman, 2002)
Cooperative and responsibility-based activities	Peer roles and shared problem solving	Social responsibility	Relatedness	Peer support; responsibility behavior	(Aygun et al., 2024; Bean et al., 2018; Beni et al., 2017; Masten, 2001; Pozo et al., 2018; Southwick et al., 2014)
Stress and emotion awareness activities	Challenge appraisal and guided reflection	Recognition of emotional responses	Coping regulation	Coping strategy use; perceived stress	(Bernstein & McNally, 2017, 2018; Folkman et al., 1986; Gross, 2002; Gross & John, 2003; Stults-Kolehmainen & Sinha, 2014)
Recovery and lifestyle modules	Recovery logs and brief planning prompts	Recovery-related awareness	Lifestyle self-regulation	Recovery plan; lifestyle reflection	(Ajzen, 1991; Cruz et al., 2024; Michie et al., 2011; Nutbeam, 2000; Schwarzer et al., 2011; Sørensen et al., 2012; Tremblay et al., 2016; Zimmerman, 2002)

**Table 3:** Priority research directions and feasible indicators for PE-based lifestyle learning.

Research Priority	Primary Construct	Feasible Indicators	Interpretation Boundary	Key Supporting Literature
Mechanism-focused PE classroom studies	Motivation and self-regulation	Brief PE motivation scale; task-specific self-efficacy; teacher observation	Short-term classroom change does not establish lifestyle transfer	(Aygun et al., 2024; Bandura, 1977; Deci & Ryan, 2000; Fernández-Río et al., 2024; Fredricks et al., 2004; Kahu, 2013; Kahu & Nelson, 2018; Lonsdale et al., 2011; Pozo et al., 2018; Ryan & Deci, 2000; Teixeira et al., 2012; Vasconcellos et al., 2020; Zhoc et al., 2019; Zimmerman, 2002)
Stress–emotion–recovery learning	Coping and recovery awareness	Brief coping measure; recovery log; student reflection	Measures capture selected processes, not overall well-being	(Bandura, 1977; Bernstein & McNally, 2017, 2018; Chaput et al., 2016; Chaudhary et al., 2020; Cohen et al., 1983; Connor & Davidson, 2003; Cruz et al., 2024; Folkman et al., 1986; Gross, 2002; Gross & John, 2003; Gullone & Taffe, 2012; Lang et al., 2016; Neumann et al., 2022; Nutbeam, 2000; Samad et al., 2024; Sørensen et al., 2012; Stults-Kolehmainen & Sinha, 2014)
Transfer beyond PE	Lifestyle self-regulation	Action plan; follow-up log; interview	Intention or self-report does not confirm sustained behavior	(Ajzen, 1991; Bean et al., 2018; Holt et al., 2017; Michie et al., 2011; Schwarzer et al., 2011; Zimmerman, 2002)
Implementation feasibility	Curriculum enactment	Fidelity checklist; lesson observation; teacher interview	Feasibility and fidelity may vary across contexts	(International Conference on Health Promoting Universities and Colleges, 2015; Kennedy et al., 2021; Lever et al., 2024; Lewallen et al., 2015; Olsson et al., 2024)

Note: Candidate indicators are illustrative rather than prescriptive. Measures should be selected according to the proposed curriculum mechanism, target population, cultural and educational context, expected time frame of change, and feasibility within typical PE settings.

## Data Availability

No new data were created or analyzed in this study. Data sharing is not applicable to this article.

## Abbreviations

The following abbreviations are used in this manuscript:

ERQ-CA	Emotion Regulation Questionnaire for Children and Adolescents
PE	Physical Education
